# Improved RAPD Method for *Candida parapsilosis* Fingerprinting

**DOI:** 10.3390/genes14040868

**Published:** 2023-04-05

**Authors:** Iwona Wojciechowska-Koszko, Magdalena Mnichowska-Polanowska, Paulina Roszkowska, Michał Sławiński, Stefania Giedrys-Kalemba, Barbara Dołęgowska, Monika Sienkiewicz, Beata Hukowska-Szematowicz, Paweł Kwiatkowski

**Affiliations:** 1 Department of Diagnostic Immunology, Pomeranian Medical University in Szczecin, 70-111 Szczecin, Poland; 2 Department of Medical Microbiology, Pomeranian Medical University in Szczecin, 70-111 Szczecin, Poland; 3 Department of Laboratory Diagnostics, Public Clinical Hospital No. 2 in Szczecin, 70-111 Szczecin, Poland; 4 Department of Laboratory Medicine, Pomeranian Medical University in Szczecin, 70-111 Szczecin, Poland; 5 Department of Pharmaceutical Microbiology and Microbiological Diagnostic, Medical University of Lodz, 90-151 Lodz, Poland; 6 Institute of Biology, University of Szczecin, 71-412 Szczecin, Poland; 7 Molecular Biology and Biotechnology Center, University of Szczecin, 71-412 Szczecin, Poland

**Keywords:** RAPD-PCR, optimization, orthogonal array, genetic diversity, epidemiology, *Candida parapsilosis*

## Abstract

Recently, methods based on the analysis of arbitrarily amplified target sites of genome microorganisms have been extensively applied in microbiological studies, and especially in epidemiological studies. The range of their application is limited by problems with discrimination and reproducibility resulting from a lack of standardized and reliable methods of optimization. The aim of this study was to obtain optimal parameters of the Random Amplified Polymorphic DNA (RAPD) reaction by using an orthogonal array as per the Taguchi and Wu protocol, modified by Cobb and Clark for *Candida parapsilosis* isolates. High Simpson’s index values and low Dice coefficients obtained in this study indicated a high level of interspecies DNA polymorphism between *C. parapsilosis* strains, and the optimized RAPD method proved useful in the microbiological and epidemiological study.

## 1. Introduction

Traditional diagnostic methods, based on analysis of the phenotypic features of fungi, do not fully respond to present requirements. Microscopic observation methods do not always give univocal results, and the culture of fungi and biochemical tests, performed for the purpose of their further identification, require quite a long incubation and are often not sufficient for epidemiological investigation [1,2]. Diagnostic difficulties with the identification of fungi on a species level have successfully stimulated the development of cognitive and applicative research. Among them, molecular methods take prominent place, making the development of modern, fast, and unequivocal identification of these microorganisms possible [1,2,3,4]. These methods enable not only species differentiation, but also show interspecies differences [5].

Genetic analysis uses the allelic variability of gene products or the variability on the level of nucleic acids. Its basic assumption is that strains proceeding from one clone show the possibility of observing DNA or the polymorphism of proteins, distinguishing them from other unrelated strains of the given species. This is especially illustrated in the method described as Multiple Arbitrary Amplicon Profiling (MAAP), which is based on the amplification of DNA with the use of arbitrary primers. In this instance, DNA polymorphism reveals the band pattern characteristic for every isolate, i.e., DNA fragments of different lengths, separated electrophoretically. Differences between strains depend statistically on the quantity of the analyzed bands [6,7].

The most-often applied methods from the MAAP group are Arbitrary Primed Polymerase Chain Reaction (AP-PCR, which uses accidental sequences starters) and Random Amplified Polymorphic DNA (RAPD; the accidental amplification of polymorphic DNA fragments) [8,9,10], which are described as “fingerprinting” methods [2,7,11,12,13]. The differences between these methods concern the length of the primers and the way the resulting products are separated. In both methods, random attachment of primers during amplification is possible by significantly lowering the temperature from 42 °C to 36 °C. In the AP-PCR method, one or two different primers with a length of 18–24 bp can be used, and the obtained amplification profile is separated on a polyacrylamide gel. On the other hand, in the RAPD method, the primers used are 10–12 bp in length, and the resulting products (from 3 to 10 bands) are separated on an agarose gel. Many experimental studies show that the discriminatory power of these methods can be increased by using two or more short primers [2,7,11,12,13].

The RAPD method described by Williams et al. [10], as with other methods from the MAAP group, does not require information about the sequence of the target DNA, as opposed to other genetic methods, such as Restriction Fragment Length Polymorphism (RFLP) [6,14,15]. Another advantage of the RAPD method is its greater discriminatory power compared to the RFLP-PCR method, where every strain actually gives a specific profile of amplification [16,17].

Thus, when choosing a system of genetic typing one must, first of all, take into account whether the method fully meets the necessary criteria. The method should reliably determine typeability and repeatability, which means the probability of obtaining the same results in repeated tests based on one sample. Furthermore, the method should have high discriminatory ability, that is, to be able to qualify strains proceeding from different sources as unique [6,7,17]. Other essential and important practical advantages of genetic methods, compared to phenotypic methods, are also the ease in performing and interpreting results and analysis at low cost [7]. However, the analysis that meets these three main criteria requires optimization of the method used. The lack of standardization in research procedures is the most frequent reason, making the repeatability of results impossible, which causes erroneous interpretation and limits the wider use of genetic methods in microorganism diagnostics [2,16,18,19,20,21].

The aim of the study was the optimization of the RAPD method to obtain the greatest possible discriminatory ability of interspecies differentiation of *C. parapsilosis* strains.

## 2. Materials and Methods

### 2.1. Yeast Strains

Thirty-nine isolates (derived from oral cavity, anus, and interdigital spaces) of *C. parapsilosis* were taken from the Chair of Microbiology, Immunology and Laboratory Medicine, Pomeranian Medical University in Szczecin (Poland) collection. These strains were described in detail in our previous study, including the biochemical and molecular identification, with the inclusion of the new species *C. orthopsilosis* and *C. metapsilosis* [22]. To extract DNA, strains were inoculated onto a solid Yeast Extracted–Peptone–Dextrose medium (bioMérieux, Warsaw, Poland) and incubated overnight at 37 °C. Then, single colonies were suspended in 2 mL liquid Tryptic Soy Broth medium (TSB, bioMérieux, Warsaw, Poland) and re-incubated for 24 h at 37 °C.

### 2.2. DNA Extraction

Genomic yeast DNA was isolated as described by Graham et al. [23], with some modifications. Yeast cells were separated from the TSB medium by centrifugation at 14,000× *g* for 7 min. Each pellet was washed with sterile de-ionized water and centrifuged at 14,000× *g* for 7 min. Then, the pellet was suspended in a 600 μL extraction buffer (1 M Tris pH 8.0, 10% sodium dodecyl sulfate) (Sigma-Aldrich, Poznan, Poland) and incubated at 100 °C for 30 min in a water bath. The lysate was centrifuged at 14,000× *g* for 7 min, and after centrifugation the supernatant was put into a new tube and phenolated three times. A mixture of phenol/chloroform/isoamyl alcohol (25:24:1; *v*/*v*/*v*) (Sigma-Aldrich, Poznan, Poland) was used in two first phenolates, and a mixture of chloroform/isoamyl alcohol (24:1; *v*/*v*) was used in the last phenolate. Then, DNA was extracted with 1/10 estimated sample volumes of 7.5 M sodium acetate (pH 5.2) (Sigma-Aldrich, Poznan, Poland), and two estimated sample volumes of 98% ethanol (Sigma-Aldrich, Poznan, Poland) at −20 °C for 1 h, and centrifuged at 14,000× *g* for 17 min. The extracted DNA was washed twice with 70% ethanol and centrifuged each time at 14,000× *g* for 7 min. The extract was dried at 37 °C for 30 min in an incubator. The dried pellet was dissolved in 100 μL of sterile de-ionized water at 37 °C for 20–24 h and was stored at −20 °C for later study.

### 2.3. Primers

A preliminary study was performed separately with six oligonucleotide primers—1247, 1290, RP2, RP4-2, SOY, and OP-AO3 (Table S1). All oligonucleotide primers were synthesized by Symbios (Gdansk, Poland). A further study was performed with primer combinations—each primer with each primer. The criterion of choice was the number of amplicons after electrophoresis for individual primers or their combinations.

### 2.4. Optimization of RAPD-PCR Reaction

Optimization was performed according to the protocol of nine reactions as presented in Table 1, suggested by Taguchi and Wu, and modified by Cobb and Clarkson [3,6,24] for variable concentrations of four components (MgCl_2_, dNTPs, primers, and DNA templates). The concentration of components was the following: component 1 (MgCl_2_: A—2 mmol/L, B—2.5 mmol/L, C—3 mmol/L), component 2 (dNTPs: A—1.5 mmol/L, B—2 mmol/L, C—2.5 mmol/L), component 3 (primers: A—10 pmol/L, B—20 pmol/L, C—30 pmol/L), and component 4 (DNA: A—10 ng/L, B—20 ng/L, C—30 ng/L).

The number of required experiments (*E*) was calculated by Equation (1):

(1) E=2k+1

where *k* is the number of tested components (in study four). These components were used in three different concentrations and applied in reactions according to orthogonal arrays, as presented in Table 1. Such a scheme provides them with an identical frequency of repetition.

The number of amplified products in each lane was counted. Their total number was determined using Equation (2):

(2) Y=r+1

where *Y* is the number of bands in the lane increased by one, and *r* is the number of bands in the next lane.

The value *Y* was used to calculate the SN_L_ coefficient according to Equation (3):

(3) $${\mathrm{SN}}_{L}=-10\log\left(\frac{1}{n}\overset{i}{\underset{i=1}{\sum }}\frac{1}{Y_{i}^{2}}\right)$$

where SN_L_ is the signal to noise ratio, n is the number of concentrations used in the study (in our study: three), and *Y* is the number of bands in the lane increased by one.

### 2.5. Calculation of Discrimination Power of RAPD-PCR

The discriminatory power (D) of the RAPD method for the *C. parapsilosis* species was determined using Simpson’s index [11] in Equation (4):

(4) $$D=1-\frac{1}{N\left(N-1\right)}\overset{s}{\underset{j=1}{\sum }}n_{j}\left(n_{j}-1\right)$$

where *N* is the total number of strains of examined species, *S* is the total number of obtained types (*j*), and *n_j_* is the number of strains belonging to a specific type (*j*).

The D value can range from 0 to 1. The values received near to 1 in this number range show the high discriminatory power of the method. The repeatability of the RAPD results was checked by carrying out two separate amplification reactions for two randomly chosen *C. parapsilosis* strains.

### 2.6. RAPD-PCR Conditions

The amplification reactions were carried out on a Gene Amp PCR System 9600 thermocycler (Perkin-Elmer, Waltham, MA, USA). RAPD-PCR conditions for all primers used included 42 cycles. The temperature and duration for each profile was the following: denaturation at 94 °C for 1 min, annealing at 36 °C for 1 min, and elongation at 72 °C for 1 min. The final elongation was run at 72 ° C for 7 min complete cycles.

The initial 25 μL PCR optimal reaction mixture contained 10 × PCR buffer (100 mmol/L Tris-HCl, 500 mmol/L KCl, pH 8.3), 1U of Taq DNA polymerase (Roche Diagnostics GmbH, Mannheim, Germany), 30 ng/L DNA template, 2.5 mmol/L MgCl_2_, 2 mmol/L dNTPs, and 20 pmol/L for each primer (RP2 and 1247).

### 2.7. Gel Electrophoresis

The amplification products, negative control, and a DNA molecular mass marker were analyzed by horizontal electrophoresis in a 2% agarose gel stained with 0.5 mg/L ethidium bromide (Sigma-Aldrich, Poznan, Poland), in a 1 × Tris-borate-EDTA running buffer at 120 V for one hour. The 1.5 kb DNA ladder (Fermentas, Waltham, MA, USA) was used as a DNA marker. DNA bands were illuminated under UV light and photographed in an image system, GelDoc-It2 Imager (Upland, CA, USA).

### 2.8. Analysis of the Amplification Results

Results were made in the BioGenProfil software (BioProfil—BioGen Windows Application Version 99.04, Glostrup, Denmark). A dendrogram was generated by the Unweighted Pair Group Method with the Arithmetic Mean (UPGMA) method. The similarity values of DNA profiles were computed based on band positions by using the Dice coefficient Equation (5):

(5) $$S_{\mathrm{AB}}=\frac{2a}{\left(2a+b+c\right)}$$

where a is the number of similar bands, and b and c are the numbers of different bands in the two lanes compared.

The S_AB_ value can range from 0 (no common bands in two compared profiles) to 1 (all bands identical). Strains were clustered using an 70% homology cut-off, above which strains were closely related and assigned to the same cluster.

## 3. Results

Figure S1 shows amplification profiles for chosen primers performed empirically for one randomly selected strain of *C. parapsilosis* (F31_T2_OC_CP). Using only one primer, three to six bands per lane were obtained, but when a combination of primers was used, the number of bands per lane increased. The highest discriminatory power for interspecies differentiation of *C. parapsilosis* was obtained with the primer combination, RP2 and 1247, which initially generated the most complex amplicon profiles (up to eight bands per lane).

**Figure 1:**
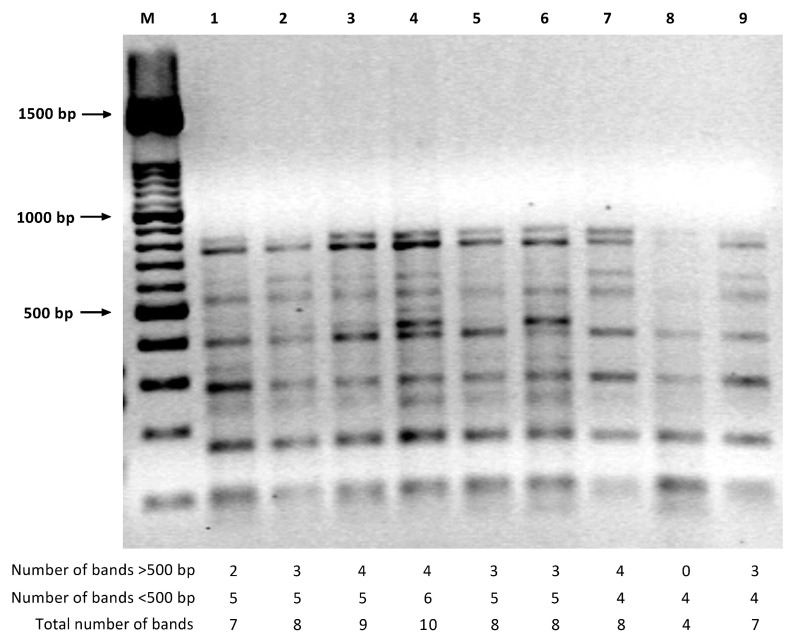
Amplification profiles for orthogonal array reactions performed for *C. parapsilosis* (F15_T1_IS_CP) and RP2 and 1247 primers. M—marker; lanes 1–9 obtained for different concentrations of four components (MgCl_2_, dNTP, primers, DNA) prepared according to the orthogonal array.

**Figure 2:**
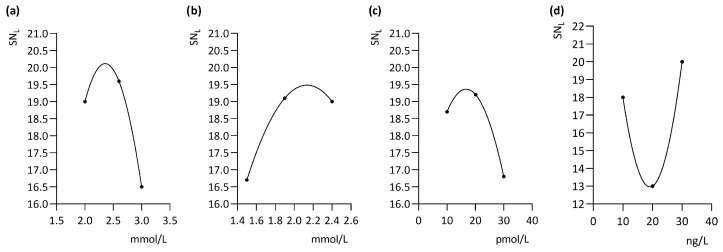
Results of particular components (**a**—MgCl_2_, **b**—dNTPs, **c**—RP2 and 1247 primers, **d**—DNA) on the signal-to-noise ratio (SN_L_) for RAPD-PCR fingerprinting and RP2 and 1247 primers.

Figure 1 shows the obtained profiles for reactions carried out in the orthogonal array according to Table 1. The mean yield for each reaction was used to calculate the SN_L_ ratio for each of the four tested components (Table 2). These were used to estimate the optimal reaction conditions by calculating individual component curves using regression analysis (Figure 2). These were determined as 20 pmol/L per primer, 30 ng/µL target DNA, 2.5 mmol/L MgCl_2_, and 2 mmol/L dNTPs.

Figure S2 shows dendrogram similarity within *C. parapsilosis* strains derived from the Dice coefficient. Thirty-nine *C. parapsilosis* strains were investigated, which were isolated from twenty-two people. Based on the dendrogram analysis, twenty-two types of band patterns were affirmed, which contained nine identical band patterns (26 strains), nine similar band patterns (S_AB_ 70–100%), and four unique (Un) band patterns. These types, except for the Un band patterns, qualified to 10 genotypes and were described from A to J on the dendrogram. The Un patterns were described from Un1 to Un4. The discriminatory power of the method used for genetic analysis of *C. parapsilosis* strains was characterized by the high Simpson’s index value, 0.92, which indicated large genetic differentiation within tested strains. The genetic analysis of *C. parapsilosis* strains will be the subject of a separate publication [22].

## 4. Discussion

The RAPD technique used in our study towers over phenotypic methods and other genetic methods in various ways, such as its speed, relatively low cost, and the possibility for it to be technically carried out in many laboratories and applied to the differentiation of many organisms [2,7,11,12,13]. However, its credibility has been questioned in terms of its repeatability [7,17]. Its application in our study’s optimization scheme, proposed by Taguchi and Wu and modified by Cobb and Clarkson, made the elimination of this disadvantage possible, permitted us to produce repeatable results, and influenced the adjustment of the quality of the amplification of profiles received.

Optimization is a very time-consuming stage, but it should be an indispensable part of every genetic research method, and our own study shows this. Optimization is most-often carried out by trial and error. Attempts to correct optimization of four variables such as MgCl_2_, dNTPs, primers, and DNA (each in three different concentrations), without any scheme would require the execution of 81 independent reactions. The lack of a scheme procedure leads to a blindfold manipulation of component concentrations of the reaction’s mixture, increasing financial expenditure, prolonging the time taken, and not always guaranteeing the achievement of satisfying and repeatable results [25]. Applying the optimization scheme proposed by Taguchi and Wu allows for the number of reactions necessary to be limited to nine, saving time and analysis and, most importantly, ensuring the credibility of the typing that is carried out.

Even small changes to the concentration of single elements of the reaction’s mixture will mutually interact and influence qualitative and quantitative differences in received electrophoretic images. This is associated with the vanishing of bands in some band profiles, or with the appearance of artifacts, often in the form of dimeric bands. Many scientists consider that the most essential in the optimization of conditions for duplication is the usage of suitable concentrations of magnesium ions and a DNA matrix [3,6,7,26]. A testing of the quantity is recommended in regard to the DNA matrix in the range 0.1–50 ng/L [1,6], although some authors consider that the most optimal range is 10–25 ng/L [7]. Usage of too-large a quantity of DNA can be seen by the appearance of smears, whereas too-small a quantity causes the disappearance of reliable bands, which gives considerable divergences in results obtained for the same strains. Quantities of DNA obtained during optimization in our study were within the range recommended by other scientists and were placed in the 10–30 ng/L range. An equally significant influence on the results received is the quantity of matrix DNA, its quality, and the concentration of magnesium ions, all of which causes an excess rise in artifacts and cost of natural products. We received a similar effect with an excess of primer concentration, which is directly related to the concentration and the quality of isolated DNA of examined microorganisms [3,6,7,26]. Additionally, authors point to differences in results with pollutions of term stable DNA polymerase (coming from an *Escherichia coli* vector or *Thermus aquaticus*), in terms of quality, origin, and concentration [6,26]. There is a very large number of variables in this method essentially influencing the outcome, and the requirements of the optimization scheme would ensure their elimination. It appears that the orthogonal array on which the Taguchi and Wu scheme is based, and which was applied to our own studies, fulfils these requirements because it takes into consideration interactions between concentrations of sub-stratums which occur in equal frequency. The important advantage of this method, proved also in our own studies, is the considerable reduction in the number of repeated experiments, which reduces the consumption of sub-stratums, saving time and contributing to a reduction in expenses of analysis [6]. In the case of our own examinations, in addition to the benefits mentioned earlier, optimal conditions of the reaction for the chosen microorganisms were obtained, and were also indispensable in the proceeding genetic typing scheme. Further to optimization, the selection of primers is a very important stage, and has a significant influence on results obtained, and the same is true for the amount of discriminatory power the given method has [27,28]. Contents of nucleotides G+C in primers chosen for the selection oscillated in a range of 40–60%, and therefore were not different from standard quantities required in the RAPD method. Additionally, the primers chosen did not possess a palindromic sequence [29]. Many experimental studies prove that the discriminatory power of the RAPD method can be increased by using two or more short primers [1,6,7,30]. A combination of two primers used in our study increased the standard of detected polymorphism in obtained genetic profiles and demonstrated an optimal image of the genetic profile, which was confirmed by a high Simpson index value. It is noteworthy that, during the genetic analysis, unique strains of *C. parapsilosis* were also identified. The primers used in combination, selected during optimization assay, generated electrophoretic profiles characteristic in terms of both the number and size of striations. In our study, for the primer combinations used, the number of striations obtained ranged from 1 to 10 and their size from 100 bp to 1.5 kb. The primer combination giving the highest number of striations in the obtained profile was selected for the final analysis. Other researchers’ studies of genetic differentiation of *C. parapsilosis* strains used primers that gave similar results [31,32,33].

Optimization of the typing procedure used in the present study is therefore a pivotal step to obtain reliable and reproducible results. It should therefore include selection of primers and evaluation of concentrations of key reaction components. The number of amplicons generated, depending on the primer–matrix interaction, must be sufficient to demonstrate heterogeneity between related but distinct fungal strains [22].

Of the six single primers and their combinations tested, primers RP2 and 1247 proved to be the most discriminating based on the number and size range of amplicons generated and the complexity of the resulting genotypes. The concentrations of primers, magnesium ions, dNTPs, and the DNA template in the reaction mixture were evaluated as critical factors determining amplification efficiency and specificity. The quality of RAPD-PCR genotypes was significantly affected by both too-high and too-low concentrations of all components tested; therefore, optimal concentrations had to be selected. The optimized method protocol provided a reliable and accurate analysis of the genetic diversity and relatedness between the *C. parapsilosis* isolates tested.

In the current study, the obtained molecular patterns showed significant genetic diversity among the tested *C. parapsilosis* strains, as most strains represented different genotypes.

The RAPD-PCR method used in this study was standardized to assess intra-species genetic diversity of *C. parapsilosis* strains, which epidemiologically play an increasingly important role in the increase in nosocomial fungal infections in hospitalized patients, especially in intensive care units [34,35]. The applicability of the above method in epidemiological investigations is confirmed, among others, by the studies of other researchers [34,35] who showed that all environmental strains identified as *C. parapsilosis* in intensive unit care units had the same RAPD pattern as *C. parapsilosis* clinical isolates. This confirms the theory of horizontal transmission of strains and indicates infection via the hands of medical personnel. This observation is therefore consistent with the general view that, in intensive care units, *C. parapsilosis* outbreaks are mainly due to deliberate disruption of the skin barrier for the administration of invasive therapies and the use of monitoring equipment [34,35].

## 5. Conclusions

Analysis of *C. parapsilosis* genetic material by means of optimized and standardized methods offers new chances in distinguishing strains. It eliminates the problem of non-standard strains, does not require species-specific reagents, and can be applied to examinations of various microorganisms. It is possible to make use of this in microbiology and epidemiology for species identification, genetic changeability studies, and human biology and phylogenetic research. Indeed, the lack of standardization of tests is the most common reason that prevents the reproducibility of results, causes misinterpretation of results, and limits the wider use of genetic methods in identification diagnosis.

## Figures and Tables

**Table 1:** Scheme of optimization of RAPD reaction according to Taguchi and Wu—orthogonal arrays for four components at three different concentrations.

Number of Reaction	Component 1	Component 2	Component 3	Component 4
1.	A	A	A	A
2.	A	B	B	B
3.	A	C	C	C
4.	B	A	B	C
5.	B	B	C	A
6.	B	C	A	B
7.	C	A	C	B
8.	C	B	A	C
9.	C	C	B	A

**Table 2:** SN_L_ ratios for a set of RP2 and 1247 primers used for *C. parapsilosis* fingerprinting.

Number of Bands (Base Pair)	Components	Concentrations of Tested Components		
		A (SN_L_)	B (SN_L_)	C (SN_L_)
>500 bp	MgCl_2_	6…6…6 (15.57)	7…6…6 (15.97)	5…5…5 (13.98)
	dNTPs	6…7…5 (15.32)	6…6…5 (14.97)	6…6…5 (14.97)
	Primers	6…6…5 (14.97)	6…7…5 (15.32)	6…6…5 (14.97)
	DNA	6…6…5 (14.97)	6…6…5 (14.97)	6…7…5 (15.32)
<500 bp	MgCl_2_	3…4…5 (11.47)	5…4…4 (12.60)	5…1…4 (4.35)
	dNTPs	3…5…1 (4.16)	4…4…5 (12.60)	5…4…4 (12.60)
	Primers	3…4…5 (11.47)	4…5…4 (12.60)	5…4…1 (4.35)
	DNA	3…4…4 (11.04)	4…4…1 (4.26)	5…5…5 (13.98)
Total	MgCl_2_	8…9…10 (19.00)	11…9…9 (**19.59**) *	9…5…8 (16.46)
	dNTPs	8…11…5 (16.71)	9…9…9 (**19.08**) *	10…9…8 (19.00)
	Primers	8…9…9 (18.73)	9…11…8 (**19.18**) *	10…9…5 (16.84)
	DNA	8…9…8 (18.39)	9…9…5 (13.17)	10…11…9 (**19.91**) *

* Optimized concentrations of the tested components are marked in bold.

## Data Availability

Data are contained within the article.

## Supplementary Materials

The following supporting information can be downloaded at: https://www.mdpi.com/article/10.3390/genes14040868/s1, Table S1: Characteristics of primers applied in this study. Figure S1: Amplification profiles for chosen primers performed for *C. parapsilosis* (F31_T2_OC_CP) in a 2% agarose gel. Figure S2: Dendrogram derived from the Dice similarity coefficient (2%) showing the relationship between tested *C. parapsilosis* (CP) isolates according to our previous study [22].
