# Supplementary material for: Improved RAPD Method for Candida parapsilosis Fingerprinting
Source: Genes (Basel). 2023 Apr 5;14(4):868. doi: 10.3390/genes14040868 (PMC10137414; doi:10.3390/genes14040868)
Supplement: Supplementary file 1 [file genes-14-00868-s001.zip › genes-2276993-supplementary.pdf]

Table S1. Characteristic of primers applied in this study.

Primer	Sequence	G+C Content	Reference
1247	5'-AAGAGCCCGT-3'	60%	[1]
1290	5'-GTGGATGCGA-3'	60%	[2]
RP2	5'-AAGGATCAGA-3'	40%	[3]
RP4-2	5'-CAGATGCTTC-3'	50%	[4]
SOY	5'-AGGTCAGTGA-3'	50%	[3]
OP-AO3	5'-AGTCAGCCAC-3'	60%	[5]

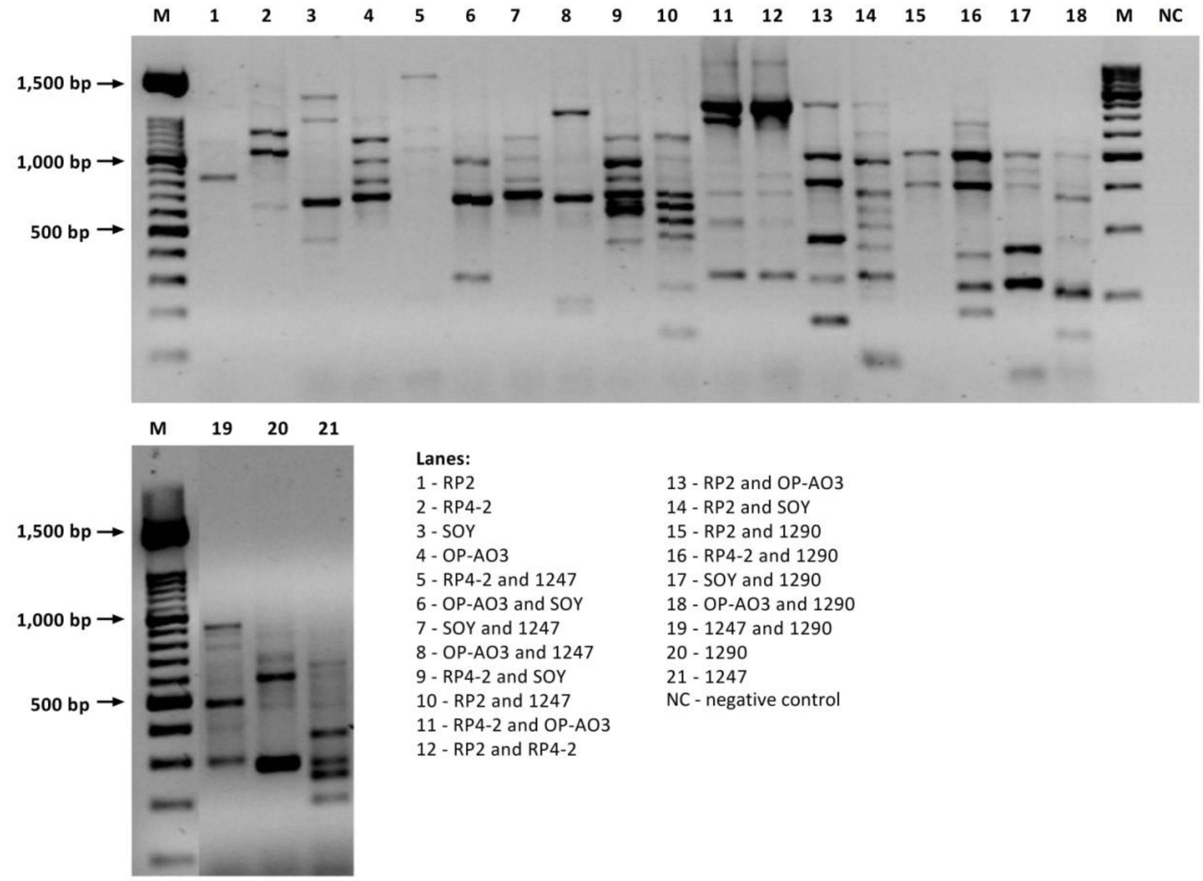

Figure S1. Amplification profiles for chosen primers performed for *Candida parapsilosis* (F31_T2_OC_CP) in a 2% agarose gel. M – marker, lanes 1-21 – amplicons obtained for different individual primers and their combinations, NC – negative control.

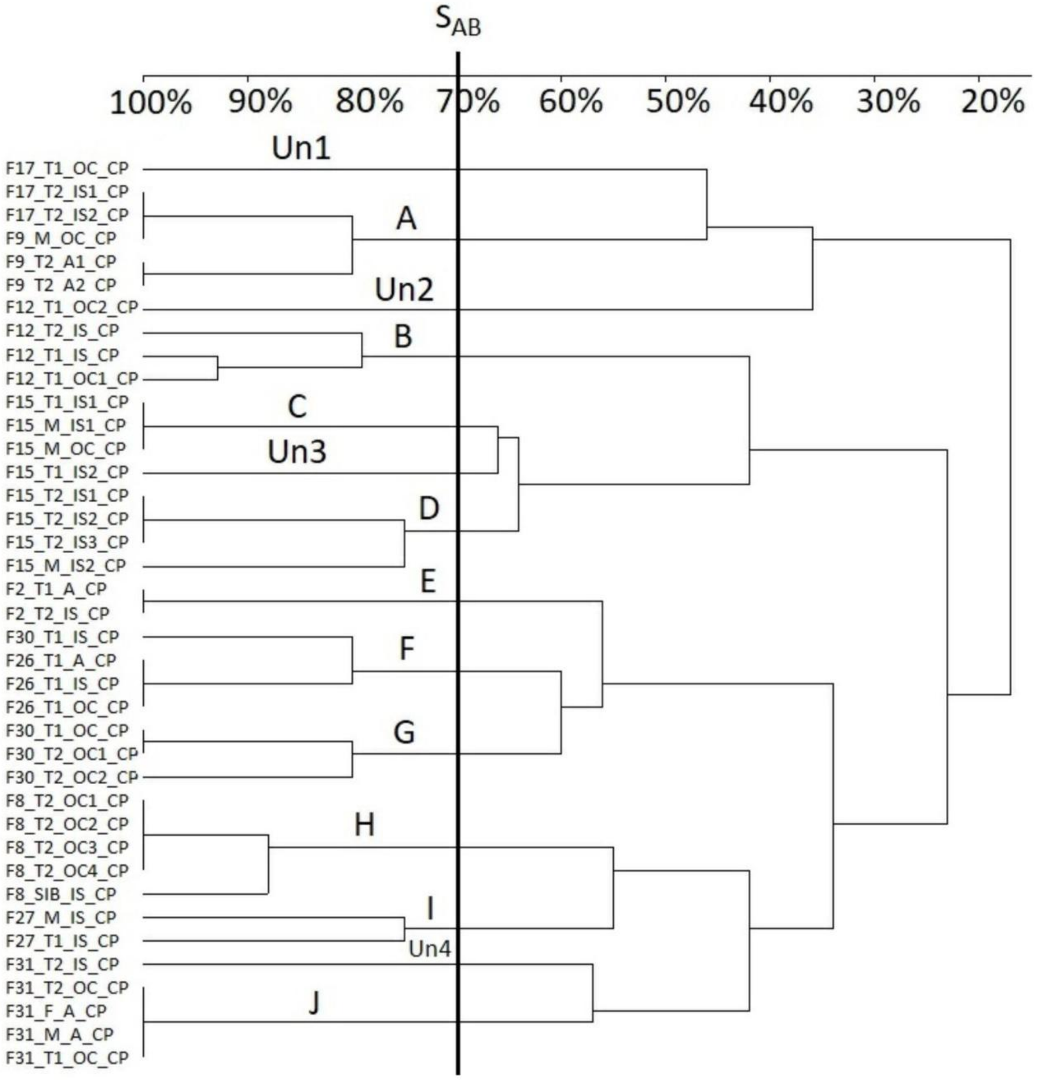

Figure S2. Dendrogram derived from the Dice similarity coefficient (2%) showing the relationship between tested *Candida parapsilosis* (CP) isolates according to our previous study [6]. Strains described according to the following pattern: number of family_member of family_source of isolation_species.

References

1. Kersulyte, D.; Woods, J.P.; Keath, E.J.; Goldman, W.E.; Berg, D.E. Diversity among clinical isolates of Histoplasma capsulatum detected by polymerase chain reaction with arbitrary primers. *J. Bacteriol.* **1992**, *174*, 7075–7079.
2. Woods, J.P.; Kersulyte, D.; Tolan, R.W.J.; Berg, C.M.; Berg, D.E. Use of arbitrarily primed polymerase chain reaction analysis to type disease and carrier strains of *Neisseria meningitidis* isolated during a university outbreak. *J. Infect. Dis.* **1994**, *169*, 1384–1389.
3. Lehmann, P.F.; Lin, D.; Lasker, B.A. Genotypic identification and characterization of species and strains within the genus *Candida* by using random amplified polymorphic DNA. *J. Clin. Microbiol.* **1992**, *30*, 3249–3254.
4. Di Francesco, L.F.; Barchiesi, F.; Caselli, F.; Cirioni, O.; Scalise, G. Comparison of four methods for DNA typing of clinical isolates of *Candida glabrata*. *J. Med. Microbiol.* **1999**, *48*, 955–963.
5. Gyanchandani, A.; Khan, Z.K.; Farooqui, N.; Goswami, M.; Ranade, S.A. RAPD analysis of *Candida albicans* strains recovered from different immunocompromised patients (ICP) reveals an apparently non-random infectivity of the strains. *Biochem. Mol. Biol. Int.* **1998**, *44*, 19–27.
6. Wojciechowska-Koszko, I.; Kwiatkowski, P.; Roszkowska, P.; Krasnodębksa-Szponder, B.; Sławiński, M.; Gabrych, A.; Giedrys-Kalemba, S.; Dołęgowska, B.; Kowalczyk, E.; Sienkiewicz, M. Genetic diversity of *Candida* spp. isolates colonizing twins and their family members. *Pathogens* **2022**, *11*, 1532.
